# Supplementary material for: Comparative Analysis of a Rapid Quantitative Immunoassay to the Reference Methodology for the Measurement of Blood Vitamin D Levels
Source: Methods Protoc. 2025 Aug 1;8(4):85. doi: 10.3390/mps8040085 (PMC12388426; doi:10.3390/mps8040085)
Supplement: Supplementary file 1 [file mps-08-00085-s001.zip › mps-3667503-supplementary.pdf]

Supplementary Table S1. Comparison Differences

Subject ID	Vitamin D levels (nmol/L)		Difference between readings	
	IgLoo	DBS	Value	%
13	20.45	7.15	13.3	186
24	12.775	7.5	5.275	70
6	18.3	7.65	10.65	139
50	15.125	8.25	6.875	83
3	19.65	12.1	7.55	62
45	35	12.8	22.2	173
44	39.75	15.8	23.95	152
17	14.325	16.8	-2.475	-15
18	26.75	17.85	8.9	50
31	31.75	18.35	13.4	73
25	35.25	21	14.25	68
34	39	22.5	16.5	73
27	30	23.15	6.85	30
4	43.75	25.2	18.55	74
10	40.75	25.25	15.5	61
23	52.75	26	26.75	103
46	50	26.35	23.65	90
30	44.25	26.95	17.3	64
11	45	27.35	17.65	65
49	41.75	28.9	12.85	44
33	61.5	29.3	32.2	110
19	54.25	30.4	23.85	78
26	38.5	30.5	8	26
5	50.75	31.3	19.45	62
37	65.25	31.45	33.8	107
41	51	32.8	18.2	55
20	37.75	34.5	3.25	9
1	31	34.65	-3.65	-11
7	60.75	45.7	15.05	33
32	69.5	47.35	22.15	47
12	66.75	47.45	19.3	41
8	98	47.5	50.5	106
36	70.75	49.05	21.7	44
21	58.25	50.3	7.95	16
14	78	50.35	27.65	55
15	63.5	50.5	13	26
47	68.25	52.55	15.7	30
40	75.25	54.45	20.8	38
38	86.75	54.7	32.05	59
48	66.25	56.9	9.35	16
42	115.25	57.15	58.1	102
9	89.5	59.5	30	50
29	116.5	59.5	57	96
28	95.75	60.55	35.2	58
16	91.75	62.85	28.9	46

2	90.5	65.7	24.8	38
22	81.5	70.85	10.65	15
39	108.5	119.1	-10.6	-9
		**Average:**	**18.8**	**62%**

Supplementary Table S2. Comparison Differences part 2

Subject ID	IgLoo Vitamin D levels (nmol/L)	DBS Vitamin D levels (nmol/L)
13	20.45	7.15
24	12.775	7.5
6	18.3	7.65
50	15.125	8.25
3	19.65	12.1
45	35	12.8
44	39.75	15.8
17	14.325	16.8
18	26.75	17.85
31	31.75	18.35
25	35.25	21
34	39	22.5
27	30	23.15
4	43.75	25.2
10	40.75	25.25
23	52.75	26
46	50	26.35
30	44.25	26.95
11	45	27.35
49	41.75	28.9
33	61.5	29.3
19	54.25	30.4
26	38.5	30.5
5	50.75	31.3
37	65.25	31.45
41	51	32.8
20	37.75	34.5
1	31	34.65
7	60.75	45.7
32	69.5	47.35
12	66.75	47.45
8	98	47.5
36	70.75	49.05
21	58.25	50.3
14	78	50.35
15	63.5	50.5
47	68.25	52.55
40	75.25	54.45
38	86.75	54.7
48	66.25	56.9
42	115.25	57.15
9	89.5	59.5
29	116.5	59.5

28	95.75	60.55
16	91.75	62.85
2	90.5	65.7
22	81.5	70.85
39	108.5	119.1
